# A Complex Combination Therapy for a Complex Disease–Neuroimaging Evidence for the Effect of Music Therapy in Schizophrenia

**DOI:** 10.3389/fpsyt.2022.795344

**Published:** 2022-03-15

**Authors:** Elena Ivanova, Tzvetina Panayotova, Ivan Grechenliev, Bogomil Peshev, Penka Kolchakova, Vihra Milanova

**Affiliations:** ^1^Psychiatric Clinic, Alexandrovska University Hospital, Sofia, Bulgaria; ^2^Department of Psychiatry and Medical Psychology, Medical University, Sofia, Bulgaria; ^3^Private Practice Psychiatrist, Plovdiv, Bulgaria

**Keywords:** schizophrenia, music therapy, combination therapy, neuroimaging, negative symptoms, cognitive deficits

## Abstract

Schizophrenia is a disease characterized by clinical polymorphism: a combination of diverse syndromes defined by differences in structure, course and outcome. The etiology and pathogenesis of this mental disorder is still not completely understood, in spite of the achievements in the fields of neuroscience, genetics, neuroimaging and others. Different treatment strategies have been developed for patients with schizophrenia, but the search for new pharmacological agents continues with the mission of achieving a more effective control over the disease manifestations (positive and negative symptoms), improvement of the patients' social functioning and quality of life. The accumulated clinical experience has revealed that drug treatment and the inclusion in various rehabilitation programs and social skills training shows promising results in these patients. In recent years a plethora of evidence has been compiled regarding the role of music therapy as a possible alternative in the combination treatment of patients with mental disorders, schizophrenia included. Thus, the purpose of this review is to present the reader with a more detailed and science-based account of the beneficial effect of music therapy on the general wellbeing of patients diagnosed with schizophrenia. To fulfill our goal, we will focus mainly on the evidence provided by modern neuroimaging research.

## Introduction

Schizophrenia is a devastating psychiatric disorder, characterized by a variety of different symptoms that are organized in several different clusters. The positive symptoms cluster is composed of delusions, hallucinations and disorganized speech and behavior. Conversely, the negative symptoms cluster encompasses deficits in the normal daily and social functioning of the patient–lack of motivation, anhedonia, social isolation and poverty of speech (1). Currently schizophrenia is diagnosed according to the criteria described in the Diagnostic and Statistical Manual of Mental Disorders, 5th edition (DSM-5) or the International Classification of Diseases, 10th edition (ICD-10). However, modern neuroimaging tools may offer a more in-depth understanding of the brain's morphological and pathophysiological abnormalities relating to schizophrenia. This search for biomarkers is an important step in the elaboration of our knowledge about the onset, course and outcome of the disease (2). Considering the heterogeneous nature of different psychopathologies, the accumulation of data from neuroimaging studies will provide a more complex view of the affected neurocircuitry. The progress that has been made in fields such as machine learning and bioinformatics is already improving the effectiveness of large-datasets analysis (3). These advances are bringing the much needed breakthrough in the elucidation of the genetic, structural and the neuromodulatory basis of schizophrenia.

Currently, the psychopharmacological treatment of schizophrenia is based on the usage of typical and atypical antipsychotics, pharmacological agents with well-established efficacy (4). When used as maintenance treatment, antipsychotic drugs prevent the relapse of the diseases (5). Antipsychotic drugs are capable of reducing the intensity of the symptoms from the positive cluster. However, negative symptoms and cognitive deficits remain the main therapeutic obstacle as antipsychotics show little to no effect on their progression (6) from a clinical standpoint, it is also important to acknowledge the side effects of antipsychotic drugs (7). Adversities include dyskinesia, obesity and greater risk of sudden cardiac death (8–10). These side effects must be considered when analyzing the lack of compliance among some groups of schizophrenic patients (11, 12). Nevertheless, antipsychotic drugs remain essential in the management of acute psychotic states and future research in this area must take into account the possibility of overcoming the burden of the above-mentioned side effects.

Regarding negative symptoms, the introduction of third-generation antipsychotics like Cariprazine gives reason for optimism. Cariprazine is an innovative antipsychotic agent as it acts as a dual D2/D3 partial agonist, with a greater affinity for D3 receptors (13, 14). This noteworthy mechanism of action, differing vastly from that of all previously discovered antipsychotics, has been confirmed through the use of positron emission tomography (PET) scans (15). Clinical trials and observational studies have shown that Cariprazine is especially effective in the treatment of patients with predominant negative symptoms (16, 17). Negative symptoms are often viewed as multidimensional and heterogeneous. In terms of the multidimensional aspects of negative symptomatology, Cariprazine has demonstrated beneficial effects with regard to key constructs underlying negative symptoms (18). Concerning heterogeneity, negative symptoms have been strongly interlinked with, and often secondary to, cognitive, depressive, positive and motor manifestations. That allows for the implication of the unique receptor profile and mechanism of action possessed by Cariprazine in the alleviation of secondary negative symptoms (19–21). These findings complement the published results, revealing improvements in predominant negative symptoms in patients given Cariprazine, independently of the amelioration registered in other symptoms known to affect negative symptoms (16). Additionally, Cariprazine's favorable safety and tolerability aids in further differentiating the psychotherapeutic agent from the older antipsychotics (22). It stands to reason that a future clinical trial, combining the application of Cariprazine with a non-pharmacological treatment strategy and neuropsychological assessment of large patient cohorts may offer new perspective on outpatient treatment strategies for negative symptoms.

One such non-pharmacological treatment strategy is the use of music to improve the information processing capacities of the brain. Music therapy has long been used as a part of combination therapy for various neuropsychiatric disorders, ranging from affective and anxiety disorders to different forms of dementia. Neuroimaging studies provide further evidence for the brain structures and neural circuits corresponding to music processing. The processing of musical stimuli increases the activity within brain structures typically associated with the affective circuits of the brain. This effect is observed in the insula, the cingulate cortex (CC), the prefrontal cortex (PFC), hippocampus, amygdala and hypothalamus (23). Moreover, music can evoke changes in the levels of important neuromodulators like dopamine, endorphins, endogenous cannabinoids and nitric oxide (24). Understanding the beneficial effect of music is impossible if not put in the context of neuroplasticity. Neuroplasticity could be defined as the adaptive structural changes occurring in the sensory, motor and associative circuits of the brain as a response to a salient environmental stimulus. These plastic changes may be related to increased volume of certain brain areas and better connectivity between regions belonging to a particular functional circuit. In the cases of pathological or traumatic alteration in the integrity of the brain tissue, neuroplastic changes may also have compensatory function, helping to reorganize the storage and utilization of sensory information (25). Neuroimaging studies have discovered the higher rates of neuroplastic changes in the brains of musicians (26). This is yet another reason why music therapy may be a suitable choice when considering an effective therapeutic intervention during the course of outpatient treatment of patients suffering from somatic, cognitive, affective or behavioral disorders (27–29). Using innovative imaging techniques like Functional near-infrared spectroscopy (fNIRS) it has been shown that music is beneficial for memory and could modulate the activity of the PFC (30). This is further demonstrated by the utilization of Functional magnetic resonance imaging (fMRI), with studies reporting induction of plasticity and changes in connectivity during the course of music therapy, which leads to improved memory, attention and executive functions (31). Overall, the enhancement of neuroplasticity through music may invigorate various neurophysiological operations of the brain, improve movement and could even positively influence our circadian rhythms (32–34).

Regarding schizophrenia, clinical trials have provided evidence for the efficacy of music therapy as part of complex treatment (35). Music therapy may have a positive influence on the willingness of the patient to cooperate with the medical staff and other mental health professionals (36). There is a lack of general agreement about the effect of music therapy on patients with positive symptoms. Some authors have provided evidence for the beneficial effect of this kind of therapy for both positive and negative symptoms (37–39), white others have failed to replicate these results regarding the positive symptoms (40). Furthemore, the notion that musical therapy may not be sufficient in providing help for the positive symptoms of schizophrenia is further reinforced by several different meta-analyses (36, 41, 42). Regardless, neuroimaging studies provide additional support for the application of music therapy in the context of schizophrenia. A recent study has compared two groups of patients—a group of participants that was treated only with antipsychotics and another group which received musical intervention regularly for a period of 1 month along with the pharmacological treatment. Using fMRI the researchers were able to demonstrate changes in the levels of connectivity between the striatum and the areas composing the default mode network (DMN) in the patients from the music intervention group (43).

Considering the foregoing evidence, the current review paper will try to provide additional arguments why music therapy is an efficient non-pharmacological strategy for improving the cognitive deficits and the general wellbeing of those who are fighting with schizophrenia. We will summarize some of the important contributions of the field of neuroimaging in order to outline the most prominent pathological features of the diseases. By doing so, we hope to create a conceptual framework for presenting the advantages that musical therapy has offered when being considered as a part of combination treatment.

## The Complexity of Schizophrenia–Looking Through the Lenses of Neuroimaging

It is becoming increasingly evident that schizophrenia is associated with deteriorating alteration in normal brain functioning and morphology. For example, the cortex of the frontal and temporal lobes in patients with schizophrenia has been shown to thin progressively (44). These changes correspond to the onset of the disease and its course. Additionally, the thinning of the cortex may be directly related to the outcome of the pharmacological treatment (44). Chronicity in the course of the disease is associated with an increasing hypofunction of the frontal cortical areas, which is in direct correlation to the observed deficiency in attention and memory observed in these patients (45). The deficits in working memory are further confirmed by meta-analysis of neuroimaging data, linking it to the abovementioned frontal hypofunction, while also reporting increased activity in the anterior cingulate cortex (ACC) and the left frontal pole (46). Another meta-analytic study demonstrates that during resting state, in schizophrenic patients, there is decreased activity of the ventromedial prefrontal cortex (vmPFC), the left hippocampus, the posterior cingulate cortex (PCC) and the precuneus is decreased. However, the bilateral lingual gyrus seems to be more active at this state (47). Considering white matter integrity, diffusion tensor imaging (DTI) studies illustrate the reduced fractional anisotropy (FA) of the fiber tracts connecting the PFC with the temporal regions when comparing schizophrenic patients with healthy control group (48, 49). Decreased FA is also observed in the corticothalamic and interhemispheric tracks, including corona radiata and corpus callosum (50).

The pathophysiological nature of schizophrenia has long been considered to be related to dysregulated dopaminergic volume transmission. Increased synthesis and abnormal dopamine release in the striatum has been described in patients with schizophrenia (51). Elevated dopamine levels at the synapses predict good therapeutic response to the pharmacological treatment (52). Neuroimaging studies using Single-photon emission computed tomography (SPECT) and Positron emission tomography (PET) have confirmed the important role of the cortical D2/D3 receptors in the treatment of schizophrenia. However, there is a growing scientific interest in the role of other dopamine receptors, like the D5 receptors located in the prefrontal cortex. It is believed that these receptors may enhance the therapeutic effect in many psychopathologies, schizophrenia included (53).

But schizophrenia is also linked to abnormal synaptic plasticity. Novel neuroimaging approaches may shed light on the pathological processes taking part on the microscopic level. For example, PET imaging using ligands to target the Synaptic vesicle glycoprotein 2A (SV2A) is an exciting new opportunity to measure synaptic density in schizophrenic patients (54). Integrating other innovative neuroimaging methods like the neurite orientation dispersion and density imaging (NODDI) into clinical research will allow for better understanding of the pathological changes in the gray matter in schizophrenia (55).

PET, MRI and fMRI techniques also have great clinical value, as they can provide additional information on the responses of the patient to the different pharmacological treatment strategies Higher levels of striatal dopamine have been detected in patients with first psychotic episode. The observed hyperdopaminergia predicts better response to pharmacological treatment (56, 57). Conversely, alteration in the volume of the gray matter and decrease of glial cells are predictors for poor responses to antipsychotic treatment (58, 59). Neuroimaging methods could also be used to predict the outcome of the application of non-pharmacological treatment. One morphological marker for this is cortical reserve (pre-treatment gray matter volume and surface areas) (60). Cortical surface area parameters and gray matter volume have been used as evidence to explain the better social functioning of patients during the 1 year period after they have participated in Cognitive Enhancement Therapy (60). Other authors have also suggested that there is a positive connection between the greater cortical reserve in the left PFC and the improvement in memory performance after undergoing cognitive strategy training (61). The greater volume of the gray matter in the PFC is linked to the better outcome of cognitive-behavioral therapy and reduction of the symptoms of psychosis (62). Thus, cortical reserve is the prerequisite for improvement in neuroplasticity and information processing. The application of combination therapy—integrating pharmacological treatment with cognitive-behavioral and other approaches—is the most effective way to take advantage of these “hidden” capacities of the brain.

The research work outlined here showcases the complex pathophysiological profile of schizophrenia. The reviewed evidence from these neuroimaging studies demonstrates that schizophrenia is a disease that cannot be explained by a single etiological concept. The pathological processes are observed on various different levels in various different regions. The fact that multiple neurotransmitter systems are affected may explain the altered functional properties of many neural circuits. Further investigation of these altered properties will be crucial for the establishment of more advanced non-pharmacological approaches to negative symptoms and cognitive deficits characterizing schizophrenia. From the clinical point of view, several important findings were highlighted. First, the considerable release of dopamine in the striatum, the greater gray matter volume, the relatively preserved amount of glial cells and the increased activity in the frontoparietal network are considered to be potential markers for better response to psychopharmacological agents. On the other hand, the conserved volume of the gray matter in the PFC is seen as a possible predictor for the outcome of patients participation in non-pharmacological therapeutic programs.

## Music Therapy for Schizophrenia - A Science-Based Approach and Possible Therapeutic Targets

Musical therapy utilizes different components like melody, timbre and harmony to promote and improve information processing and the general wellbeing of the participant, putting the focus on the interaction between him and the therapist (63, 64). There is a growing interest toward the role of music therapy in treating different psychopathologies (65, 66). Researchers are providing evidence for the role of different musical interventions in inducing plastical changes (67). Music activates a large-scale bilateral network composed of frontal, temporal, parietal, cerebellar and limbic structures. This network processes various types of information and is involved in such cognitive domains like declarative memory, working memory language, attention, etc. (68). This ability of music to activate simultaneously numerous different brain regions makes it a perfect foundation for the development of rehabilitation strategies that target the cognitive, emotional and motor deficits associated with different neurological and psychiatric diseases. Thus, music therapy appears suitable for patients of various age groups, from children and adolescents with pervasive developmental disorders to adults and seniors suffering from stroke, Parkinson's disease and dementia (68).

Blood and Zetore (69) were the first to use PET to track cerebral activity during the exposure to pleasant music. The researchers were able to detect changes in the Regional cerebral blood flow (rCBF) while the participants were listening to extracts of favorite musical pieces. The exposure to pleasant music led to increased rCBF in the ventral striatum, the orbitofrontal cortex (OFC), the insula and the ACC, while decrease of rCBF was registered in amygdala, hippocampus and the ventromedial prefrontal cortex (vmPFC). The following fMRI studies further confirmed that there is an increase in the activity of the above mentioned limbic and paralimbic structures (32, 70, 71).

Salimpoor et al. (72) have demonstrated that dopamine is associated with the experience of pleasure related to music. Using PET the researchers have revealed that there is an increase in the dopamine released in the dorsal and ventral striatum while listening to pleasant music. This effect was most pronounced in the right caudate and the right nucleus accumbens (NA). Moreover, taking advantage of the better temporal resolution of fMRI the authors were able to observe the temporal dynamics of this reward signal. They demonstrated that while the participants were anticipating the peak of the emotional response the BOLD signal was stronger for the right caudate, in contrast to when they were actually experiencing that peak the signal shifted to the right NA.

Additional evidence for the significance of dopamine for the impact of music listening on reward comes from a recent publication by Ferreri et al. (73). The authors created a double blind within-subject pharmacological design, in which 27 healthy participants were assigned to receive orally levedopa (dopamine antagonist), risperidone (dopamine antagonist) or placebo in the form of lactose. The participants were exposed to self-selected and experimenter-selected musical excerpts. To measure the intensity of the pleasure response of the participants, the researchers examined their electrodermal activity (EDA—a marker for physiological arousal) and ask them to rate their experiences. The motivational responses of the participants were also measured. The attained results report that while levodopa enhanced the ability of the participants to experience pleasure related to music, risperidone had the opposing effect. This was also confirmed by the EDA rates of the two group when compared with placebo. Since risperidone is known to be an antagonist for the D2 receptors, it can be speculated that those receptors are crucial for the positive affective states induces by music. Moreover, the study by Ferrari et al. (73) offers an interesting perspective regarding schizophrenia, as one of the well-established negative symptoms of the disease is anhedonia—the inability to experience pleasure. Future studies should considered the effect of music therapy on anhedonic behavior and neuroimaging studies may provide further evidence for the role D2 receptors in music-evoked reward.

Beside dopamine, endogenous opioids may also be related to reward. According to Berridge and Kringelbach (74) “hedonic hotspots” within the NA contain opioid receptors that are activated when we are experiencing reward. Furthermore, while dopamine may be responsible for the anticipation of the reward, opioids may be related to actual feeling of pleasure (75). The pleasant feelings evoked by music are related to the greater activation of the ventral striatum (32).

**Figure 1:**
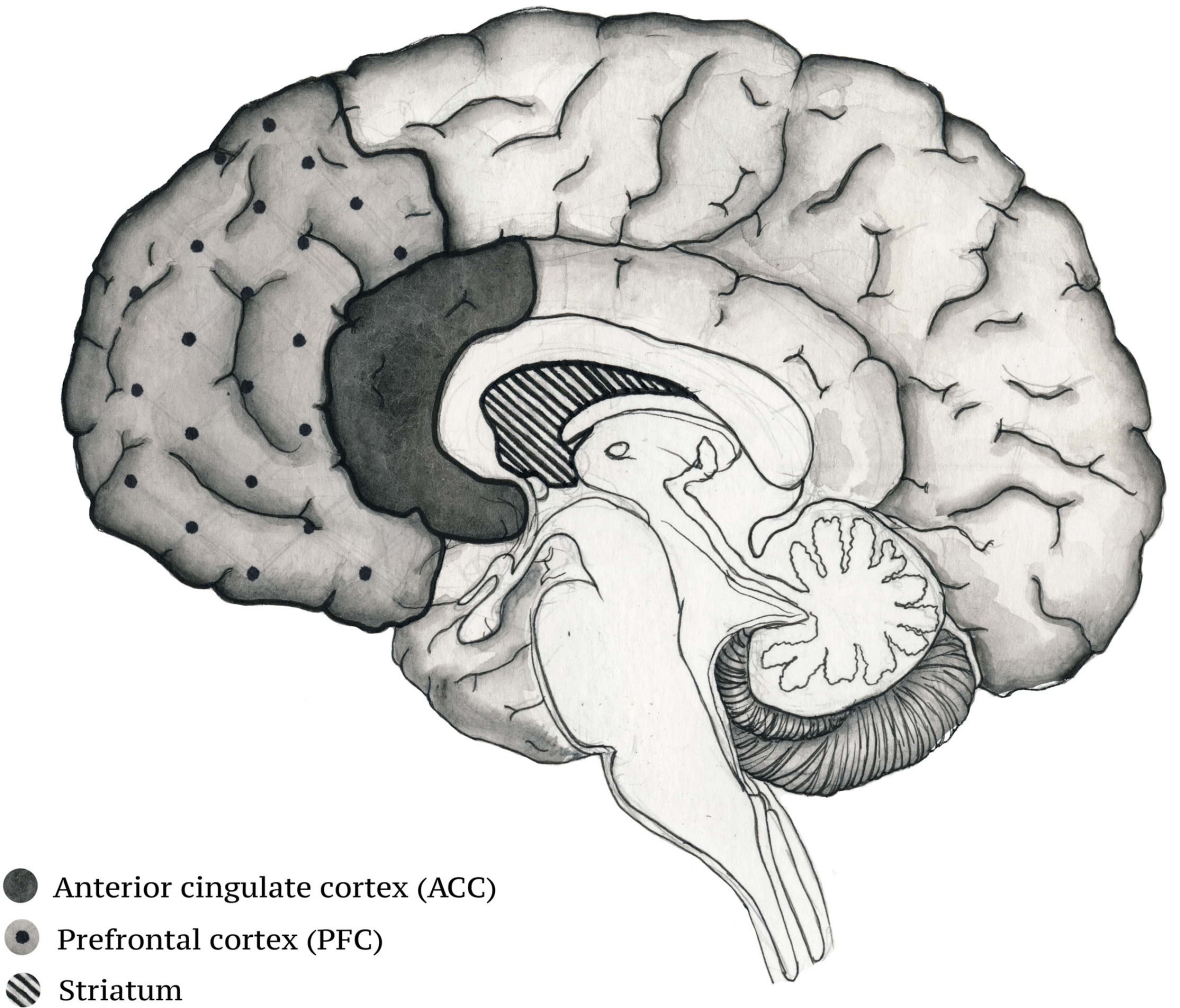
Brain structures activated during musical experience that participate in the control of homeostatic regulation.

It has been shown that some musical stimuli are processed in a laterized fashion—the stimuli that induce the feeling of tenderness activate the right ventral striatum, whereas those inducing happiness activate the left ventral striatum (71). The activation of the ACC and the insula by pleasant music may modulate homeostasis due to the projections these cortical areas send to the nuclei of the hindbrain (76). The ACC and the insula are thought to be part of a circuit responsible for the generations of feelings (77). Thus, these cortical areas are responsible for the interaction between emotional states and homeostatic control (76). The hippocampus, a structure involved in episodic memory, is responsible for the formation of memories for events associated with a particular musical piece (78). Other studies have reported that there is an increase in hippocampal activity when listening to pleasant music (79). Just like the ACC and the insula, the hippocampus can also influence homeostasis through connections with other subcortical structures (80). The effect of music on these brain areas is particularly relevant to schizophrenia as the disease has been associated with autonomic dysfunctions (Figure 1).

A study by Schultz and colleagues (81) was the first one to try to establish existing abnormalities in the interaction between the central nervous system (CNS) and the autonomic nervous system (ANS) in paranoid schizophrenic patients. By analyzing vital parameters like blood pressure, heart rate and electroencephalogram and comparing them to those of a healthy control group, the researchers were able to demonstrate the disturbed central—automatic coupling. One meta-analysis has described that the heart rate variability in schizophrenia may be linked to the dysfunction of the “top-down” control managed by the cortico-subcortical pathways that influences the activity of the brainstem where the automatic responses are initiated (82). This correlates well-with the observed hypofunction of the PFC in schizophrenia (83). All of the above evidence is in accordance with the neurovisceral integration model of Thayer and Lane (84), a model that offers a physiological link between attention, the affective functions of the brain and automatic regulation. Thus music may act to improve the synchronization of the various nodes of the cortical system dedicated to the regulation of the homeostatic states of the body.

**Figure 2:**
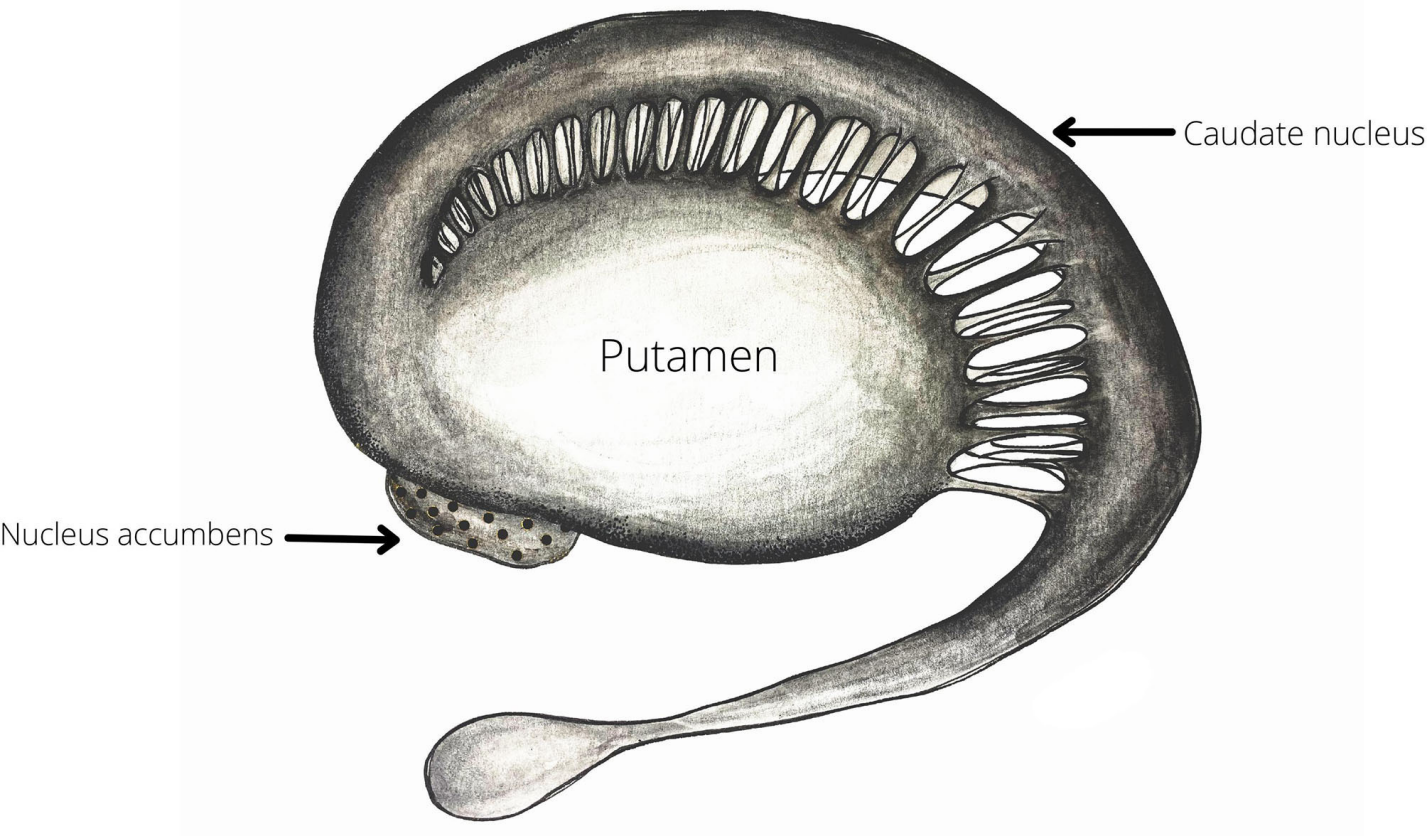
The striatum—as demonstrated by Salimpoor and colleagues (72) both the ventral (Nucleus Accumbens) and the dorsal (Caudate nucleus and Putamen) deviasions of the structure are important for music-induces reward and expectation. Therefore, the striatum may be one of most robustly activated structures during by musical exercises (Table 1).

The paper by Salimpoor et al. (72) discussed above demonstrates the pivotal role of the striatum (both caudate nucleus and nucleus accumbens) in reward anticipation and positive valence emotions. Beside this role in reward processing and motivation, the striatum has also been found to participate in the processing of temporal information (85) (Figure 2). Both animal models and human neuroimaging research have provided substantial evidence for the involvement of the striatum in interval timing (86–88) and have put forward the hypothesis that the structure is a part of a larger cortico-striatal circuit dedicated to temporal processing (89). Interval timing is the processing of temporal information in the milliseconds to seconds range. Interval timing can be further subdivided into explicit and implicit timing. Explicit timing is related to the estimation of duration and can be perceptual—stimulus duration or interstimulus interval—and motor—timing of the motor response based on the time span of the stimulus. Conversely, implicit timing is the utilization of temporal information for constructing a goal-directed behavior. Explicit timing can best be explained in terms of predictions. For example, when we try to estimate the likelihood of something happening in our environment based on the information about the duration of the available stimuli (85).

**Figure 3:**
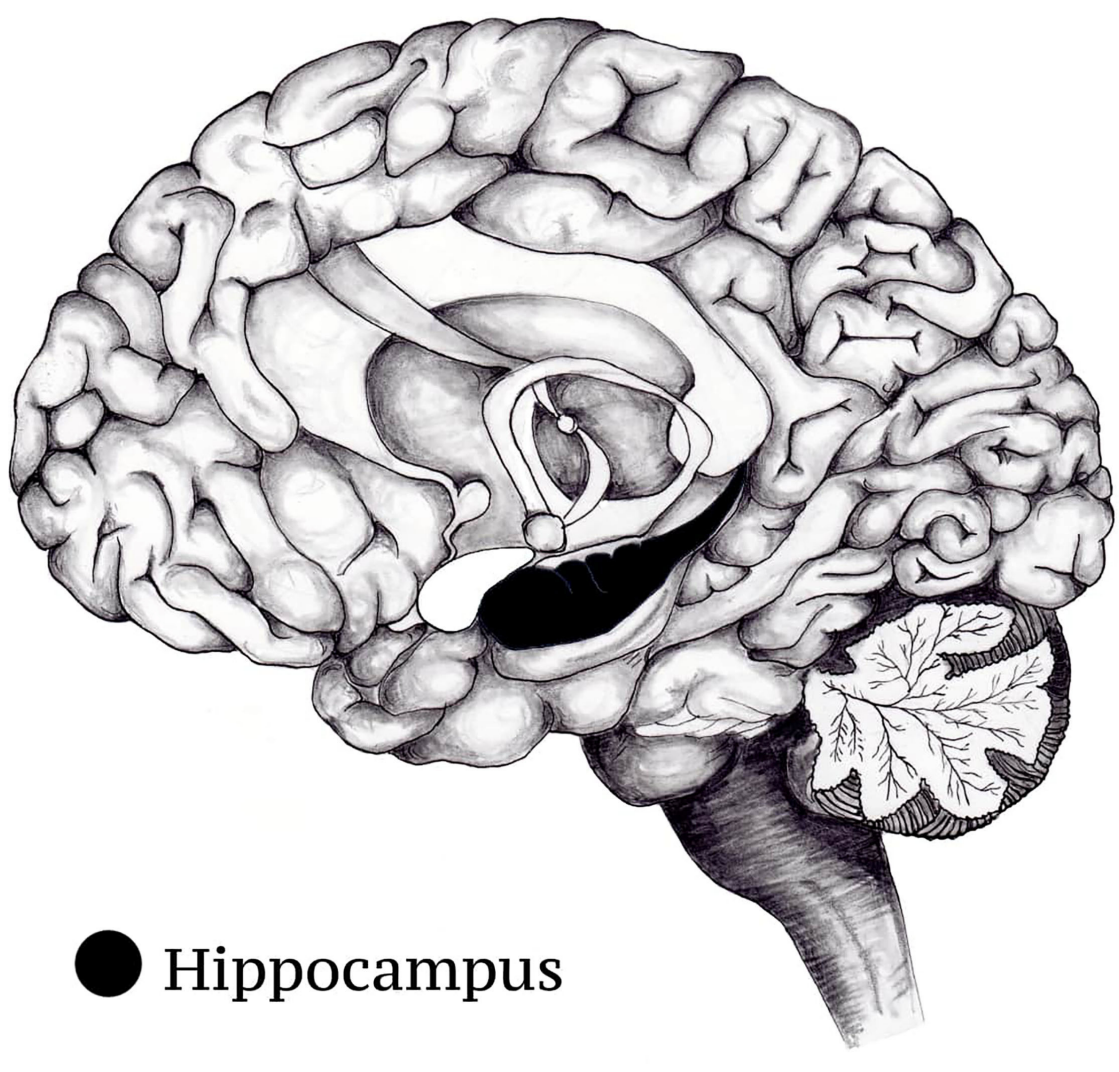
Location of the hippocampus.

Nevertheless, interval timing in the cortico-striatal circuit is not the sole form of temporal coding in the brain. Time is an important element of the contextual framework that constitutes our memory for events (episodic memory) (90). As it was already outlined, the hippocampus is the brain structure that is responsible for the formation of episodic memories (Figure 3). This requires the binding of different objects and social stimuli with the information about the spatiotemporal context in which they were encountered (91). Recently it has been discovered that groups of cells in the hippocampus fire at particular moments in between successive events to bridge them and create a unifite sequential representation. These cells came to be known as “time cells” (92). Studies using intracranial microelectrode recordings in surgical epilepsy patients have confirmed the existence of cells with similar coding strategy in the human hippocampus (93, 94).

The progression of schizophrenia has been associated with a range of cognitive impairments (95). Unsurprisingly, temporal processing is one of the many affected cognitive domains (96). Ward and colleagues (97) have reviewed the behavioral evidence related to distorted interval timing in schizophrenic patients and have suggested that a greater understanding of this deficit could bring a more detailed perspective about disease-related cognitive dysfunction in general. In addition, the authors have provided a valuable neurobiological framework for the role of D2 receptors of the striatum in abnormal interval timing. Indeed, neuropharmacological studies have advanced the knowledge about the neuromodulatory functions of dopamine in interval timing (98, 99). Considering the fact that the episodic memory system is also damaged in schizophrenia (95), it will be interesting to further elucidate how this affects temporal processing. At the current moment, evidence from the field of neuropsychology is scarce, but a recent study by Malek et al. (100) has revealed that patients with schizophrenia have difficulties locating and ordering personal events in time. The processing of temporal information in the hippocampus is drawing great attention in the field of neuroscience (101), but how schizophrenia is altering these mechanisms remains to be established.

Popov (102) has provided a number of empirical observations on how temporal processing is altered in schizophrenia. According to him, people could perceive and analyze time on two different functional levels—the psychological and the biological. Psychological time is defined as the subjective awareness, perception and estimation of physical time and its duration. As such, it does not necessarily reflect an “intrinsic sense” of time, as much as it is merely a function of different psychophysiological and psychological processes. The author further proposes that at the psychological level humans could spontaneously process events from multiple different time points as long as these events carry their own “temporal tags” in the stream of consciousness. Thus, due to the fact that events from multiple different time points are interacting in our minds, we experience changes both sequentially and simultaneously. Additionally, Popov asserts that the same time interval may be interpreted as having different duration depending on whether it concerns a currently occurring event (prospective judgment) or one that has already passed (retrospective judgment). Popov describes the phenomenon of “excessive time retention” associated with schizophrenia—the tendency of schizophrenic patients to display significantly lengthened retrospective judgment (compared to physical time), with a higher ratio of retrospective to prospective judgment, termed the coefficient of retention, showing a positive correlation with onset of schizophrenia—acute schizophrenic patients possess 3 times higher coefficients of retention as compared to subacute patients. The description of such phenomena as “excessive time retention” could allow for the deepening of current knowledge regarding the pathogenesis and diagnosis of schizophrenia, in light of the cognitive deficit observed in patients with the disorder.

By providing a brief summary of the mechanism of temporal processing in the brain and how these mechanisms could be affected by schizophrenia, we are trying to establish a novel possibility for future neuroimaging investigation of the effect of music therapy. As it was outlined already, music can enhance the synchronization between the cortical and subcortical structures that compose the cognitive and affective circuits of the brain. Timing and temporal associations could be seen as just another aspect of this synchronized activity, with structures like the striatum and the hippocampus acting as hubs that coordinate the integration and distribution of the sensory information within the circuit. According to this view, cognitive deficits and negative symptoms will be nothing more than the manifestation of desynchrony. Thus, by taking into account the clinical evaluation of the patient, the music therapist can select from a range of available exercises (summarized in Table 1) to indirectly improve the balance between the different regions of the corresponding circuit. Sometimes a more creative approach may be required—improvisation. During improvisation the patient is placed in a situation, where he or she must quickly make a series of decisions regarding timbre, rhythm, sound volume and other characteristics of the musical instrument of his choice. Conversely, the choice of musical instrument aids the patient in receiving appropriate feed-back through the produced sound. The positive effects of improvisation are associated with an improved capability for independent decision-making by the patient. The music therapist follows and records the “choices” made by the patient, as well as stimulate or “lead” (non-verbally, through improvisation in a duet or specific exercises) the patient, if the therapist finds it necessary to signal the need for making more balanced decisions.

**Table 1:** Summary of different forms of exercises that could be applied during musical therapy.

**Active group exercises**	**Performance techniques**	**Result–expected (or reported)**
Rhythmic (tactile and body moving type)	Direct finger-and-palm drumming on the surface of a hand drum. The exercise can also include feet tapping. A variety of rhythmic structures are used presenting different levels of gradually increasing complexity and speed.	Attempt to keep in synchron with the rest of the group; achieving better concentration; enhancing short-term memory through learning and reproducing short rhythmic patterns; working on the perception of time and self-awareness.
Logical rhythmic patterns overlapping with short individual “turns”	A simple regular rhythmic pulse is given to the group by the therapist to keep with unchanged. A different short rhythmic structure is introduced to a single member of the group (or improvised by him or her) to combine with the regular pulsation. The “solo” pattern is passed from one to another in a pre-set logical order or through eye contact.	Receiving and giving individual attention in a secure (friendly) environment with the active support by the therapist and the rest of the group. The individual turn is meant to take place while keeping secondary concentration into the common regular group pulsation.
Mathematical logic patterns	Rhythmic pattern set by the therapist with graded complexity. It is produced mostly using small percussive instruments and/or sound-accompanied body movements—feet and knee tapping. It can also incorporate counting and simple calculation.	Supporting logical (mathematical) thinking; development of reasoning and rationalizing of time and proportions; focus on the present moment (the “here and now”); improving short-term and working memory.
Active eye contact-based exercises	Passing of a short randomly-improvised sound signal from one participant to another through eye contact (fully non-verbal communication technique).	Stimulating visual concentration and fast reaction, eye contact and non-verbal communication; fighting eye contact fear in a friendly medium.
Simultaneous mirror exercises	Simultaneously, the movement and sound of a musical instrument are used by two –one participant is a “leader” and the other is a “performer,” watching for simultaneous performance; the “leader” and the “performer” then change their roles.	Improving visual concentration; stimulating some executive functions; various analyzers are involved.
Creative exercises	Discussion and reproduction of feelings and emotions; free conscious choice of instrument, volume and rhythmic structures; group emotional improvisation.	Stimulates personal communication and discussion and expression of feelings in a secure environment; Boosting self-confidence and understanding others' feelings.
Improvisation	Choice of instrument, its timbre, pitch and volume usage combined with the type of body movement all based on a pre-set theme to work on. The participant is given complete freedom concerning the choice of expression through sound and rhythmic structures.	Improves the ability to make independent decisions, action plans and putting them to practice. Self-awareness of the result arises by the instant answer through the sound. Improvisation itself is a process of constant decision making with respect to a wide range of details.
Emotional self-control	Training for gradual transition from one emotional state to another while improvising on a musical instrument; working with negative emotions through the sound and subsequent relaxing improvisational technique.	Helps to share and deal with negative emotion experiences transferring the awareness of the ability to daily routine.
Relaxing techniques	Reproducing relaxing timbre, body movement and breathing exercises.	Mind and body relaxation; muscle tension relief; breath and pulse regulation.

Having outlined the known and the hypothetical effects of music therapy on information processing in the brain, it is of great clinical interest to further assess the possibility of implementing a complex therapy (medication and music therapy) in treating patients with schizophrenia. Adequate assessment of this possibility necessitates the examination of certain relevant factors, such as the selection of an appropriate group of patients, choice of accompanying pharmacotherapy, number and duration of sessions, as well as evaluation of the “dose-response” relationship, when administering music therapy. With respect to inclusion criteria, researchers place emphasis on the following: age between 18 and 65 years; ICD-10 diagnostic criteria for schizophrenia being met; psychiatric examination using Positive and Negative Syndrome Scale (PANSS) with emphasis on negative symptoms, mainly blunted affect, difficulties in establishing rapport and social withdrawal. Similarly, the following exclusion criteria may be used: relatively recent onset of schizophrenia; clinical presentation dominated by positive symptoms; changes in medication in the prior month; recent hospitalization; history of substance abuse; history of significant adverse effects of antipsychotic therapy (extrapyramidal symptoms, sedation, etc.) (103). Meta-analytical results appear to corroborate the proposed idea of a beneficial influence of music therapy on symptom reduction, including negative symptoms, as well as on quality on life (104). Additionally, there is published research that suggests that music therapy may be suitable for patients with chronic schizophrenia, on account of the often coexisting deficits in verbal communication, coupled with the fact that music therapy does not necessarily depend on patient's verbal communication ability (105).

Following the above-noted definition of suitable inclusion/exclusion criteria, the attention turns to the determinants of the appropriate “dose” of music therapy as applied to schizophrenic patients, with the number of sessions varying from 7 to 78. For this reason it is necessary for the effects of music therapy to be evaluated within a short-term and mid-term timeframe (1–4 months). It is the view of the authors that adequate evaluation of the outcome of music therapy entails careful consideration of the following: the initial patient selection; session quality, as opposed to simply the number of sessions; deeper analysis of long-term effects of music therapy; the dose-response relationship (106). Alternatively, other authors have suggested complex therapy consisting of 25 sessions of music therapy, with improvements in general functioning and alleviation of negative symptoms having been reported (103). Still other findings suggest an optimal duration of 3 months for music therapy in order for an effect on negative symptoms to be registered (104). However, a more thorough investigation of the psychophysiological bases of music therapy will bring a more comprehensive view on how to best integrate and apply it as part of a complex treatment for schizophrenia. This could only be achieved by the implementation of the multidisciplinary approach in carefully controlled research settings. The greater deal of the current neuroimaging studies have been conducted with patients who have a chronic course of the disease and have been undergoing a long-term antipsychotic treatment (107–111). This brings certain limitations to the interpretation of data as to whether the obtained results are due to the progression of the disease or due to prolonged intake of antipsychotic medication. In future, it will be interesting to use neuroimaging tools to track the effect of music therapy in patients who are past their first episode of psychosis. This information will allow the music therapists to design more accurate and patient-centered programs for their clients.

## Conclusion

Music is a versatile art form, capable of evoking memories, emotions, as well as the corresponding feelings. These effects of music are related to changes in our physiology and behavior. Therefore, music could influence the interaction between our mental and homeostatic states. Here we presented evidence of different natura why music therapy is a good alternative for a non-pharmacological therapy that could be combined with pharmacological treatments to form a more efficient approach toward schizophrenia. It appears that musical therapy is more suitable for the targeting of negative symptoms associated with the disease and that it can improve to a greater extent the quality of life of the patients. This view is strongly supported by a recent meta-analysis (104). It may be that the presence of positive symptoms limits the efficacy of music therapy as the patients are experiencing deficits in attention and motor coordination. It is important to note that music therapy is well-accepted among patients and that it does not demand any prior training (112). Another advantage of music therapy, compared to other psychological interventions, is that it does not involve verbal communication. Considering that one of the negative symptoms of schizophrenia is incoherent speech, music therapy may provide an alternative way for these patients to express their thoughts and emotions (113).

In schizophrenic patients, music therapy may be viewed as complementary to pharmacotherapy (114), with its utilization as part of a complex therapy for patients with negative symptoms still being debated (115). One of the key roles that music therapy may be hypothesized to serve in the alleviation of negative symptoms, might be through synergistic effects, when coupled with psychopharmacological agents with D2/D3 partial agonistic properties, such as Cariprazine. Published research has juxtaposed the effects of stimulating and blocking agents at the D2 receptor site in terms of the resulting influence on the ability of experiencing musical pleasure, with D2 antagonists, such as risperidone, displaying considerable impairment in hedonic and motivational responses, thus potentially hindering negative symptom reduction (73). The possibility of assessing such synergism between music therapy and D2/D3 partial agonists, as mediated by changes in activity specifically at D3 receptors, is currently severely limited by the scarcity of research on the topic. Further investigation on these D3 receptor specific mechanisms would greatly benefit the understanding of the intricacies of combination therapy use with regard to reducing negative symptomatology.

## Author Contributions

All authors listed have made a substantial, direct, and intellectual contribution to the work and approved it for publication.

## Conflict of Interest

The authors declare that the research was conducted in the absence of any commercial or financial relationships that could be construed as a potential conflict of interest.

## Publisher's Note

All claims expressed in this article are solely those of the authors and do not necessarily represent those of their affiliated organizations, or those of the publisher, the editors and the reviewers. Any product that may be evaluated in this article, or claim that may be made by its manufacturer, is not guaranteed or endorsed by the publisher.
